# Starting a conversation about estimands with public partners involved in clinical trials: a co-developed tool

**DOI:** 10.1186/s13063-023-07469-9

**Published:** 2023-07-06

**Authors:** Suzie Cro, Brennan C Kahan, Akshaykumar Patel, Ania Henley, Joanna C, Paul Hellyer, Manos Kumar, Yasmin Rahman, Beatriz Goulão

**Affiliations:** 1 Imperial Clinical Trials Unit, Imperial College London, London, UK; 2 MRC Clinical Trials Unit at UCL, London, UK; 3 Critical Care and Perioperative Medicine Research Group, Queen Mary University, London, UK; 4 HEALTHY STATS Public Partner Co-Chair with Imperial Clinical Trials Unit, Imperial College London, London, UK; 5 HEALTHY STATS Public Partner with Imperial Clinical Trials Unit, Imperial College London, London, UK; 6 Health Services Research Unit, University of Aberdeen, Aberdeen, UK

**Keywords:** Clinical trial, Estimand, Patient and public involvement

## Abstract

**Background:**

Clinical trials aim to draw conclusions about the effects of treatments, but a trial can address many different potential questions. For example, does the treatment work well for patients who take it as prescribed? Or does it work regardless of whether patients take it exactly as prescribed? Since different questions can lead to different conclusions on treatment benefit, it is important to clearly understand what treatment effect a trial aims to investigate—this is called the ‘estimand’. Using estimands helps to ensure trials are designed and analysed to answer the questions of interest to different stakeholders, including patients and public. However, there is uncertainty about whether patients and public would like to be involved in defining estimands and how to do so. Public partners are patients and/or members of the public who are part of, or advise, the research team. We aimed to (i) co-develop a tool with public partners that helps explain what an estimand is and (ii) explore public partner’s perspectives on the importance of discussing estimands during trial design.

**Methods:**

An online consultation meeting was held with 5 public partners of mixed age, gender and ethnicities, from various regions of the UK. Public partner opinions were collected and a practical tool describing estimands, drafted before the meeting by the research team, was developed. Afterwards, the tool was refined, and additional feedback sought via email.

**Results:**

Public partners want to be involved in estimand discussions. They found an introductory tool, to be presented and described to them by a researcher, helpful for starting a discussion about estimands in a trial design context. They recommended storytelling, analogies and visual aids within the tool. Four topics related to public partners’ involvement in defining estimands were identified: (i) the importance of addressing questions that are relevant to patients and public in trials, (ii) involving public partners early on, (iii) a need for education and communication for all stakeholders and (iv) public partners and researchers working together.

**Conclusions:**

We co-developed a tool for researchers and public partners to use to facilitate the involvement of public partners in estimand discussions.

**Supplementary Information:**

The online version contains supplementary material available at 10.1186/s13063-023-07469-9.

## Background

Clinical trials are conducted to evaluate how well healthcare interventions work. However, in trials, as in routine health practice, treatments are not always taken by patients exactly as prescribed [1, 2]. When this happens, by looking at trial data in various ways, different (and potentially more relevant) questions can be addressed such as: ‘does the treatment improve health outcomes for all patients even if it is not taken as instructed?’ or ‘does the treatment improve health outcomes only for patients who take treatment as prescribed?’ Because the answers to different questions can sometimes lead to alternative conclusions on treatment benefits, when planning a trial, it is important to have clarity on exactly what questions the trial needs to address [3, 4]. The trial can be subsequently designed, conducted, and analysed to ensure the key clinical questions of interest are addressed.

To facilitate clarity on precisely what a trial intends to investigate, international trial regulatory guidelines (ICH E9(R1)) call for trialists to clearly define the treatment effects being investigated by using *estimands* [5]. An estimand is a precise description of what treatment effect a trial is aiming to address (for examples, see [3, 6]). It describes what the numerical result obtained in the trial will mean. By clarifying the interpretation of numerical results, estimands can help ensure these results are relevant and useful to all stakeholders, including patients and public. Since the question of most relevance to address will likely vary across different stakeholders, more than one question of interest, hence estimand, may need to be investigated in a trial.

There is increasing attention being placed on using estimands in trial design, therefore giving patients and public the possibility of getting involved in this critical part of trial design is important. Previous work has identified that patients and public want to be involved in determining numerical aspects of trials [7, 8]; however, there is uncertainty about whether they would like to be involved in discussing and defining estimands as this has not been previously explored. Moreover, there is first a need to break down the barriers of scientific jargon and explain what an estimand is to public partners. Public partners are patients and/or members of the public who are part of the research team or advise the research team (not trial participants). We aimed to (i) co-develop a practical tool with public partners that describes what an estimand is and what impact it may have in trial results interpretation and (ii) explore public partners’ perspectives on discussing estimands with public partners when designing a trial.

## Methods

An online consultation meeting was held with researchers and public partners from an established statistical trial methodology project, the HEALTHY STATS public involvement group, in January 2022. The HEALTHY STATS public involvement group was initiated in January 2021 to provide advice on an NIHR funded project to develop accessible statistical methods to determine treatments effects that matter to patients and prescribing physicians in randomised controlled trials (Advanced fellowship NIHR300593). The specific remit of the HEALTHY STATS public involvement group was to support the researchers to improve the information reported from clinical trials for patients and public. The group consisted of five public partners (AH, JC, MK, PH, YR) who were chosen to represent the patient and public voice. Members were aged between 20 and 70 years of mixed ethnicities and gender, and the group was co-led by SC and AH. Four researchers with statistical expertise facilitated the breakout discussions (SC, BK, AP, and BG). All attendees were located in England or Scotland.

Prior to the meeting a practical two-page tool to explain what an estimand is and why it matters was first drafted by members of the research (SC, BK, and BG) team. This was based on knowledge of the estimand framework described in ICH E9(R1) [5] and the researchers previous clinical trial experience.

The specific objectives of the meeting were to:

1. Co-develop a practical tool with public partners that the study team can use to help explain to public partners involved in trial planning what an estimand is and what impact it may have on trial results.
2. Explore public partners perspectives on discussing and defining the precise treatment effects to be found out (i.e. estimands) with public partners when designing a trial

The online meeting used the Zoom platform and was audio-recorded and lasted 2 h. Public partners’ perspectives were collected using Zoom polls and open-ended questions. After the meeting, the poll responses on usefulness of the tool were tabulated and the audio-recording was transcribed by SC. Discussion points were sorted into four general topic areas by SC and BG.

The practical tool describing estimands was reviewed and further developed by the public partners during the meeting. Afterwards the tool was refined, and additional feedback was sought via email in two rounds of refinement.

## Results

### Tool to explain an estimand

All 5 of the HEALTHY STATS group voted that the tool was useful. The feedback obtained on the tool included the following: (i) public partners found the tool useful to start a discussion about what question a trial is answering in a trial design context; (ii) they recommended the use of storytelling, analogies and visual aids; (iii) it was felt that the tool should be shared and a chance provided to discuss it with the trial team/statistician; (iv) public partners raised that potential trial participants might need/want to know about the estimand of the trial; however, this tool would not be indicated for that. They reported that there is already a lot of complex information presented to trial participants in patient information sheets and felt this information on estimands could be too much.

**Fig. 1:**
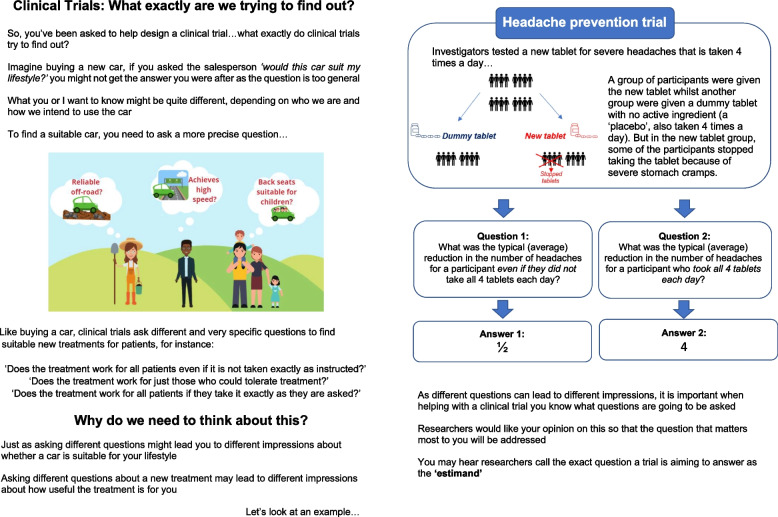
Overview of estimand explainer tool

After the meeting, the tool was updated using the feedback in an iterative process with further feedback sought via email in two rounds of refinement. Figure 1 is a screenshot of the finalised tool which explains what an estimand is and why it is important. The full tool can be found in Additional file 1 for download and use, and if you are interested in being contacted to provide feedback on the tool you can download it and provide your contact details here https://www.statsci.co.uk/ppi [9]. The tool starts with an analogy of buying a new car and explains why individuals need to ask clear and specific questions about a car to understand whether it will perform as desired for them, as different individuals have different needs. This illustrates how when testing a new treatment all stakeholders need to be clear about the precise question being asked, so that the answer is meaningful and useful.

Page two then proceeds to further demonstrate why this matters. This introduces a fictional two-group trial setting comparing a new tablet against a dummy (placebo) treatment for headache prevention. It explains how a group of trial participants were given the dummy treatment whilst another given the new tablets which were to be taken four times a day, but how some participants in the new tablet group stopped taking treatment early because of stomach cramps. It illustrates how asking different questions in this trial can lead to different results and therefore why it is important to think carefully about which specific question we want to know the answer to.

In the headache trial, by asking question one—what was the typical reduction in the number of headaches for a participant even if they did not take all tablets each day?—there is an average difference of half a headache between the groups. By asking question two—what was the typical reduction in the number of headaches for a participant who took all 4 tablets each?—there is an average difference of 4 headaches between the groups.

The tool ends by bringing in the estimand term as a label to refer to the exact question (i.e. treatment effect) the trial is aiming to answer.

### Public partner perspectives

**Fig. 2:**
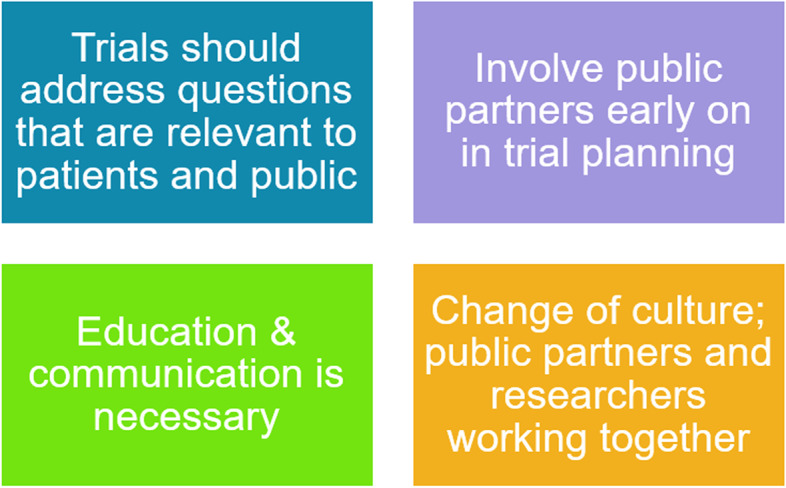
Four key topics raised by public partners on discussing the precise research question addressed in a trial

The four key topics that came out of the public partner discussions are summarised in Fig. 2.

#### Topic 1: Trials should address questions that are relevant to patients and public

Public partners highlighted the importance of conducting trials that matter to patients and public. To achieve this, they raised how trials need to select outcomes that matter to the patient. They understood that we could find out the answer to many different questions in a single trial for the same outcome and felt we should be asking patients what they want from the trial. Public partners expressed that they would want a say on this, particularly if it is public funded research. They acknowledged we cannot always serve everyone and directly address all patient and public desires, but by engaging with public partners, researchers can identify their needs and ensure trials get close to what matters most to them.

#### Topic 2: Involve public partners early on in trial planning

To make sure trials give answers relevant to patients, it was felt important to involve public partners early on in initial trial planning meetings, e.g. in trial steering committee meetings. If this is not done early on, then there was a concern the public partner voice may be overlooked later. By involving early on, there is opportunity to shape the direction of the trial.

#### Topic 3: Education and communication is necessary

To achieve successful involvement in this area, education and communication from researchers was felt necessary as this is a new area unfamiliar to patients and public. They acknowledged this would take time and be labour intensive to educate people to know how to ask the right questions.

#### Topic 4: A change of culture is required of public partners and researchers working together

Public partners indicated it will require a change of culture of researchers and public partners working closely together to achieve this. They felt talking about the precise scientific question addressed in a trial is a complex issue for a lay person. There is a need to break barriers between researchers and public partners by ensuring lay terms are used and to make it clear why the precise definition of the research question is important. Along with discussing the precise research question in general, we considered the estimand term which is the label used to describe the precise definition of the treatment effect. Initially, the word estimand was felt to be a piece of ‘statistical jargon’ that was ‘uninteresting’ to know. However, after discussion, public partners felt that if researchers are going to use this term around them, for example in trial steering committee meetings involving other researchers, they do need to first explain what it means. The co-developed tool can be described to achieve this (Fig. 1).

## Discussion

### Main findings

Overall, we found there was unanimous support from the HEALTHY STATS group to involve public partners in discussions around choosing estimands. Public partners want a say on the precise research question a clinical trial addresses so that trials do find out what matters to patients and the public. If investigators are going to use the estimand term in trial design conversations involving public partners, this term needs to be explained. We have co-developed a practical tool with the HEALTHY STATS group that explains what an estimand is and why it matters, to facilitate discussions during trial planning. Researchers can share and discuss this with public partners involved in trial design, to start a conversation around estimands.

### Research in context

Our findings demonstrate a desire for public partners to be involved in setting the precise research question addressed in a trial analysis, which is line with previous research that has identified patients and public wanting to be involved in other numerical aspects of clinical trials [7, 8]. The topics we found via discussion with the HEALTHY STATS public partners (Fig. 1) are in line with previous literature. For example, the communication and jargon challenge and need for education to enable meaningful patient and public involvement in research and especially around statistics [7, 10, 11]. The importance of conducting involvement early in the research process has also been recommended before [12, 13]. The calls for change of culture to enable better patient and public involvement are also highlighted in a recent survey, where public partners identified the main barrier for involvement to have an impact is tokenism and not being taken seriously [14].

A previous overview of systematic reviews of patient and public involvement in clinical trial design concluded that involvement can be beneficial but requires resources, preparation, training, flexibility and time [15]. The review highlighted a need for clarity within a common language which our public partners also clarified with respect to the estimand. We hope that the developed tool provides such clarity in this emerging area.

### Strengths and limitations

This is, to our knowledge, the first workshop discussing estimands with public partners, and it addresses a main barrier to involvement in clinical trial statistics [7, 11] by introducing a tool to enable better communication. Our methodology has been described to show researchers how patient involvement can work in a trial methodology setting. The nature of the workshop and process was participatory and allowed everyone involved to have a say on the tool and shape its content and format. The fact that we went to the HEALTHY STATS group can also be seen as a strength: this group has its roles and expectations, and they clearly trusted their opinions would be heard in a non-judgemental way. The public partners included in our study had a mix of ages, genders and ethnicities. However, due financial restrictions, we were limited to 5 public partners and so results might not be generalisable. Although the example trial used within the tool is a drug trial, it can be used across medical disciplines to explain the concept of an estimand, for example including surgical settings and therapeutic settings. The intervention and medical condition in the example on page 2 of the tool could be easily edited and adapted by researchers to aid communication in non-pharmaceutical settings. Estimands are applicable in clinical trials across medical fields.

### Future research

The co-developed tool can be used to start a conversation about estimands with researchers and public partners, for example in trial steering committee meetings. The next steps will be to use this tool with larger different groups of public partners and in different trial design contexts and to evaluate performance and implementation. We welcome readers who use the tool to contact the corresponding author of this article (SC) to provide feedback on its implementation.

Defining an estimand typically consists of specifying five components: the population of patients targeted, the treatment conditions being compared, how intercurrent events are being handled, the outcome variable and the population level summary measure. Understanding these individual 5 aspects was beyond the scope of this project which explored the general concept of an estimand. The best way to define estimands with public partners in a trial design context needs to be established.

Public partners’ interest in being involved in estimands could be further explored, since we only asked the HEALTHY STATS group. Further research is also required to establish whether estimand information should be made available for individuals considering taking part in a trial who are different to public partners. It is currently unclear whether this information is necessary and, if so, how? We have only considered communicating about what an estimand is with public partners involved in trial design and started the conversation in this area. We have not considered whether trial participants need estimand information available, which will involve different considerations.

## Conclusions

Our experience with the HEALTHY STATS public involvement group indicates public partners want to be involved in establishing estimands early on during the planning of a clinical trial. This is to help ensure trials address what patients and the public want to know. To facilitate the involvement of public partners in estimand discussions, we co-developed a tool explaining the concept of an estimand and why it matters, which is available for researchers and public partners to use.

## Supplementary Information

**Additional file 1**.

## Data Availability

All data and materials are included in this publication.

## Abbreviation

ICH E9(R1): International council for harmonisation of technical requirements for pharmaceuticals for human use addendum on estimands and sensitivity analysis in clinical trials to the guideline on statistical principles for clinical trials
