# Supplementary material for: Starting a conversation about estimands with public partners involved in clinical trials: a co-developed tool
Source: Trials. 2023 Jul 6;24:443. doi: 10.1186/s13063-023-07469-9 (PMC10324181; doi:10.1186/s13063-023-07469-9)
Supplement: Supplementary file 1 — Additional file 1. [file 13063_2023_7469_MOESM1_ESM.pdf]

# Clinical Trials: What exactly are we trying to find out?

So, you've been asked to help design a clinical trial…what exactly do clinical trials try to find out?

Imagine buying a new car, if you asked the salesperson *'would this car suit my lifestyle?'* you might not get the answer you were after as the question is too general

What you or I want to know might be quite different, depending on who we are and how we intend to use the car

To find a suitable car, you need to ask a more precise question…

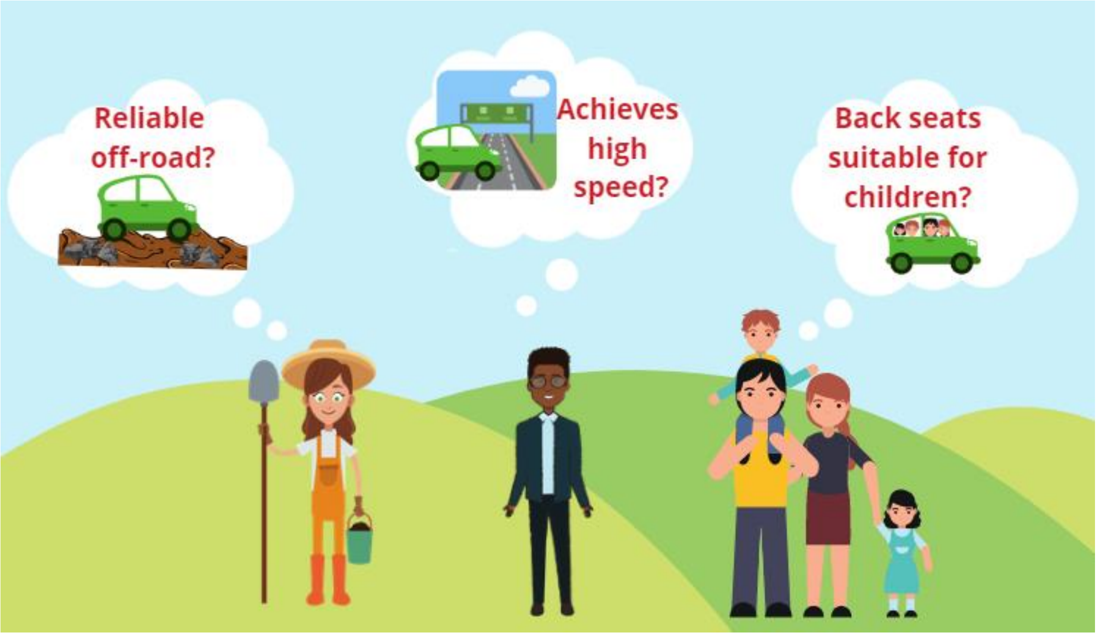


Like buying a car, clinical trials ask different and very specific questions to find suitable new treatments for patients, for instance:

'Does the treatment work for all patients even if it is not taken exactly as instructed?'
'Does the treatment work for just those who could tolerate treatment?'
'Does the treatment work for all patients if they take it exactly as they are asked?'

## Why do we need to think about this?

Just as asking different questions might lead you to different impressions about whether a car is suitable for your lifestyle

Asking different questions about a new treatment may lead to different impressions about how useful the treatment is for you

Let's look at an example…

# Headache prevention trial

Investigators tested a new tablet for severe headaches that is taken 4 times a day…

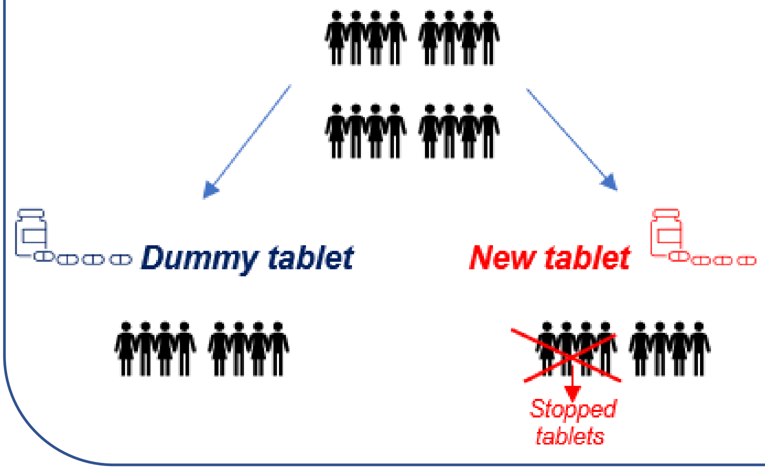


A group of participants were given the new tablet whilst another group were given a dummy tablet with no active ingredient (a 'placebo', also taken 4 times a day). But in the new tablet group, some of the participants stopped taking the tablet because of severe stomach cramps.

**Question 1:**
What was the typical (average) reduction in the number of headaches for a participant *even if they did not* take all 4 tablets each day?

**Answer 1:**
½

**Question 2:**
What was the typical (average) reduction in the number of headaches for a participant who *took all 4 tablets each day*?

**Answer 2:**
4

As different questions can lead to different impressions, it is important when helping with a clinical trial you know what questions are going to be asked

Researchers would like your opinion on this so that the question that matters most to you will be addressed

You may hear researchers call the exact question a trial is aiming to answer as the **'estimand'**
